# Phase I trial in participants with rheumatoid arthritis and healthy volunteers with CIT-013, a first-in-class extracellular traps targeted therapy

**DOI:** 10.1016/j.ero.2026.03.018

**Published:** 2026-04-28

**Authors:** Micha N. Ronner, Naomi B. Klarenbeek, Salah Hadi, Leonie M. Middelink, Maarten C. Kraan

**Affiliations:** 1 Centre for Human Drug Research, Leiden, the Netherlands; 2 ICON Netherlands B.V., Groningen, the Netherlands; 3 Citryll BV, Oss, the Netherlands

Aberrant extracellular trap (ET) formation and accumulation in tissue is associated with the pathophysiology of many inflammatory disorders, including rheumatoid arthritis (RA), as the enzymes and histones present in ETs are highly cytotoxic and proinflammatory [1].

Data suggest that neutrophils from patients with RA exist in a proinflammatory state and are primed to form ETs [2]. In addition, serum DNase from patients with RA fails to degrade ETs as efficiently as serum from healthy individuals. This is due to either polymorphisms in DNase, which render the enzyme less active or due to steric hindrance contributed by autoantibodies bound to ETs [3]. Consequently, markers for ETs, such as citrullinated histones H2a, H3, and H4 (citH2a, citH3, and citH4), are elevated in RA serum when compared with healthy individuals and are significantly higher in patients having high-to-moderate disease activity than those with mild disease activity or Remission [4].

We report data of a phase 1 trial (EudraCT number: 2020-005848-36) with a humanised monoclonal antibody (CIT-013) targeting citH2a and citH4. CIT-013 accelerates the clearance of ETs by macrophages and inhibits ET formation, thus lowering ET tissue burden with significant anti-inflammatory consequences [5,6].

We conducted a prospective, randomised, double-blind, placebo-controlled, phase I trial in 9 participants with RA and 3 healthy volunteers (HVs) (See also CONSORT Diagram in Figure). After obtaining informed consent and completing baseline assessments, either placebo, 25 mg, or 50 mg of CIT-013 was administered subcutaneously (s.c.) at days 1 and 15. Endpoints at day 29 were safety, tolerability, and pharmacokinetics (PKs). In patients with RA, the effect on citH3 and disease activity score (DAS), as measured from tenderness and swelling in 28 joints and C-reactive protein (CRP,DAS28-CRP), was followed until day 56.

**Figure:**
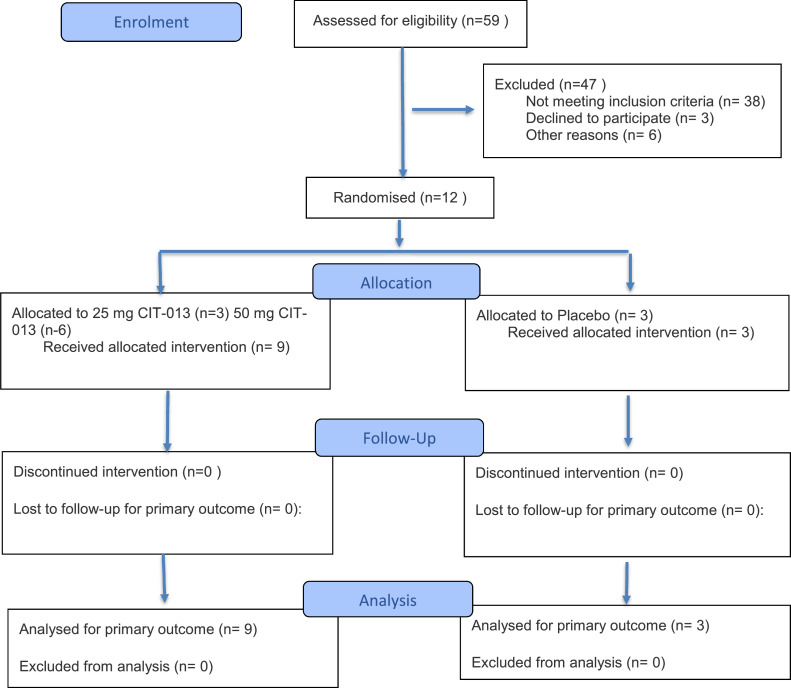
CONSORT 2025 flow diagram. Flow diagram of the progress through the phases of a randomised trial of 2 groups (that is, enrolment, intervention allocation, follow-up, and data analysis).

After randomisation, 3 participants received a placebo, 3 participants received 25 mg CIT-013, and 6 participants received 50 mg CIT-013. All 9 participants with RA met the 2010 ACR/EULAR (American College of Rheumatology, European Laegue Against Rheumatism) criteria and were on stable synthetic disease modifying antirheumatic drugs (sDMARDs) (Table). Two participants with RA received a placebo, 3 received 25 mg CIT-013, and 4 received 50 mg CIT-013.

**Table:** Clinical characteristics and mean DAS28-CRP ± standard deviation at baseline and at day 29 and DAS28-CRP response status at day 29 for the included participants with RA.

Cohort	n	Age (y)	Gender	RF+	CCP+	Disease duration (y)	Concomitant DMARD therapy	Disease status at baseline	n	DAS28-CRP baseline	DAS28-CRP day 29	No DAS28-CRP response day 29	DAS28-CRP response remission day 29
Placebo	2	50,59	1F/1M	2	2	2,7	1 MTX+HCQ, 1HCQ	Remission	1	1.6	1.7	1	0
								Active	1	5.4	4.6	1	0
25 mg	3	59,61,61	3F	1	2	10,15,36	1 MTX, 2 HCQ	Remission	3	1.9 ± 0.3	1.9 ± 0.9	3	0
50 mg	4	21,58,64,65	3F/1M	3	2	2,5,6,9	1 MTX+HCQ, 2 MTX, 1 SASP	Remission	1	2.3	3.4	1	0
								Active	3	4.2 ± 0.6	2.4 ± 0.7	0	3

F, female; HCQ, hydroxychloroquine; M, male; MTX, methotrexate; SASP, sulfasalazine; DMARD, disease modifying antirheumatic drug.

In general, CIT-013 s.c. was well tolerated. Reported adverse events were mild (grade 1) injection site reactions (all resolved spontaneously) and headache. There was 1 serious adverse event in a placebo participant with RA who required hospitalisation and surgery for a fracture. Antidrug antibodies were observed at day 56 in 2 participants with RA, 1 in the 25 mg group (titer <1-fold dilution), and 1 in the 50 mg group (titer 4-fold dilution). Both semiquantitations are considered to be low.

The half-life of CIT-013 was approximately 6 days, and all PK parameters were comparable between HV, participants with RA in remission, and those with active disease.

At baseline, 4 of 9 participants with RA (44%) had active disease, defined as a DAS28-CRP of 2.6 or higher. One participant with active RA received a placebo and continued having active disease, 3 participants with active RA received 50 mg CIT-013, resulting in a clinically relevant reduction in disease activity, reaching disease remission (n = 1), a reduction of 1.2 points in their DAS28-CRP score (n = 1), or both (n = 1). The clinical parameters for all 9 participants with RA are captured in the Table.

No HV and only 1 participant with RA with active disease (8% of total, 25% of participants with active RA) had detectable serum citH3 levels at baseline. In this participant, the level dropped below the lowest limit of detection for the duration of the study after the first 50 mg dose. Future trials will inform whether this can be a biomarker for the prediction of treatment response to CIT-013.

In conclusion, s.c. CIT-013 was well tolerated, and a DAS28-CRP reduction was observed in all participants with RA with active disease. These findings support further clinical development.

## CRediT authorship contribution statement

**Micha N. Ronner:** Writing – original draft, Validation, Resources, Project administration, Methodology, Investigation, Data curation. **Naomi B. Klarenbeek:** Writing – original draft, Validation, Supervision, Methodology, Investigation, Formal analysis, Conceptualization. **Salah Hadi:** Validation, Project administration, Investigation. **Leonie M. Middelink:** Writing – original draft, Visualization, Validation, Supervision, Resources, Project administration, Methodology, Investigation, Formal analysis, Data curation, Conceptualization. **Maarten C. Kraan:** Writing – review & editing, Writing – original draft, Project administration, Methodology, Investigation, Formal analysis, Data curation, Conceptualization.

## Competing interests

MCK, MMR, NBK, SH, and LMM report financial support and equipment, drugs, or supplies from Citryll BV. MCK and LMM also report employment and equity or stocks in Citryll BV. MMR and NBK report employment with the Foundation Center for Human Drug Research. SH reports employment with ICON Clinical Research LLC.

## References

[1] Khandpur R., Carmona-Rivera C., Vivekanandan-Giri A., Gizinski A., Yalavarthi S., Knight J.S. (2013). NETs are a source of citrullinated autoantigens and stimulate inflammatory responses in rheumatoid arthritis. Sci Transl Med.

[2] Bach M., Moon J., Moore R., Pan T., Nelson J.L., Lood C. (2020). A neutrophil activation biomarker panel in prognosis and monitoring of patients with rheumatoid arthritis. Arthritis Rheumatol.

[3] Zervou M.I., Andreou A., Matalliotakis M., Spandidos D.A., Goulielmos G.N., Eliopoulos E.E. (2020). Association of the DNASE1L3 rs35677470 polymorphism with systemic lupus erythematosus, rheumatoid arthritis and systemic sclerosis: structural biological insights. Mol Med Rep.

[4] Peng W., Wu S., Wang W. (2023). Correlation of serum citrullinated histone H3 levels with disease activity in patients with rheumatoid arthritis. Clin Exp Rheumatol.

[5] Chirivi R.G.S., van Rosmalen J.W.G., van der Linden M., Euler M., Schmets G., Bogatkevich G. (2021). Therapeutic ACPA inhibits NET formation: a potential therapy for neutrophil-mediated inflammatory diseases. Cell Mol Immunol.

[6] van der Linden M., Kumari S., Montizaan D., van Dalen S., Kip A., Foster M. (2023). Anti-citrullinated histone monoclonal antibody CIT-013, a dual action therapeutic for neutrophil extracellular trap-associated autoimmune diseases. MAbs.

